# Supplementary material for: A Conserved Requirement for RME-8/DNAJC13 in Neuronal Autolysosome Reformation
Source: bioRxiv. 2023 Feb 28:2023.02.27.530319. Preprint. [Version 1] doi: 10.1101/2023.02.27.530319 (PMC10002637; doi:10.1101/2023.02.27.530319)
Supplement: Supplement 5 [file NIHPP2023.02.27.530319v1-supplement-5.pdf]

bioRxiv preprint doi: https://doi.org/10.1101/2023.02.27.530319; this version posted February 28, 2023. The copyright holder for this preprint (which was not certified by peer review) is the author/funder, who has granted bioRxiv a license to display the preprint in perpetuity. It is made available under aCC-BY 4.0 International license.

Supplementary Table 1: *C. elegans* Strains used in this study

	Genotype	Figure
RT3799	pwSi113 [pmec-7mScarleti RAB-7 (genomic) let858]	1
RT3972	pwSi113 [pmec-7mScarleti RAB-7 (genomic) let858]; *rme-8(b1023ts)*	1
RT3970	pwSi113 [pmec-7mScarleti RAB-7 (genomic) let858]; *rme-8(pw22[N861S])*	1
RT4061	pwSi225[Pmec-7 LMP-1 mNeonGreen let858 3'UTR]	1
RT4063	pwSi225[Pmec-7 LMP-1 mNeonGreen let858 3'UTR]; *rme-8(b1023ts)*	1
RT4056	pwSi222[Pmec-7 LMP-1 mScarlet let858 3'UTR]	1
RT4058	pwSi222[Pmec-7 LMP-1 mScarlet let858 3'UTR]; *rme-8(b1023ts)*	1
RT4634	pwSi222[Pmec-7 LMP-1 mScarlet let858 3'UTR]; pwSi495[Pmec-7 RME-8oxGFP let858]	1
RT4088	pwSi161[Pmec-7 mNeonGreen LGG-1(cDNA)let858 3'UTR]; pwSi222[Pmec-7 LMP-1 mScarlet let858 3'UTR]	2
RT4089	pwSi161[Pmec-7 mNeonGreen LGG-1(cDNA)let858 3'UTR]; pwSi222[Pmec-7 LMP-1 mScarlet let858 3'UTR]; *rme-8(b1023ts)*	2
RT3969	pwSi161[Pmec-7 mNeonGreen LGG-1(cDNA) let858 3'UTR]	2

bioRxiv preprint doi: https://doi.org/10.1101/2023.02.27.530319; this version posted February 28, 2023. The copyright holder for this preprint (which was not certified by peer review) is the author/funder, who has granted bioRxiv a license to display the preprint in perpetuity. It is made available under aCC-BY 4.0 International license.

RT3971	pwSi161[Pmec-7 mNeonGreen LGG-1(cDNA) let858 3'UTR]; *rme-8(b1023ts)*	2
RT3970	pwSi161[Pmec-7 mNeonGreen LGG-1(cDNA) let858 3'UTR]; *rme-8(pw22[N861S])*	2
RT4077	pwSi161[Pmec-7 mNeonGreen LGG-1(cDNA) let858 3'UTR]; *snx-1(tm847)*	2
RT4581	pwSi225[Pmec-7 LMP-1 mNeonGreen let858 3'UTR]; *vps-15(or1235ts)*	3
RT4580	pwSi225[Pmec-7 LMP-1 mNeonGreen let858 3'UTR]; *bec-1(ok691) IV/nT1 [qIs51]*	3
RT4582	pwSi225[Pmec-7 LMP-1 mNeonGreen let858 3'UTR]; *dyn-1(ky51ts)*	3
RT4583	pwSi225[Pmec-7 LMP-1 mNeonGreen let858 3'UTR]; *epg-8(bp251)*	3
RT4499	pwSi225[Pmec-7 LMP-1 mNeonGreen let858 3'UTR]; *atg-18(gk378)*	3
RT4644	pwSi225[Pmec-7 LMP-1 mNeonGreen let858 3'UTR]; *epg-1(bp414)*	3
RT4645	pwSi225[Pmec-7 LMP-1 mNeonGreen let858 3'UTR]; *epg-6(bp242)*	3
RT4637	pwSi222[Pmec-7 LMP-1 mScarlet let858 3'UTR]; pwSi495[Pmec-7 RME-8oxGFP let858]; *rme-8(b1023ts)*	5
RT4635	pwSi495[Pmec-7 RME-8::GFP let858]; pwSi503 [Pmec-7 mScarleti SNX-1 cDNA let858]	5
RT4573	pwSi161[Pmec-7 mNeonGreen LGG-1(cDNA) let858 3'UTR]; *vps-15(or1235ts)*	6
RT4500	pwSi161[Pmec-7 mNeonGreen LGG-1(cDNA) let858 3'UTR; *bec-1(ok691) IV/nT1 [qIs51]*	6

bioRxiv preprint doi: https://doi.org/10.1101/2023.02.27.530319; this version posted February 28, 2023. The copyright holder for this preprint (which was not certified by peer review) is the author/funder, who has granted bioRxiv a license to display the preprint in perpetuity. It is made available under aCC-BY 4.0 International license.

RT4574	pwSi161[Pmec-7 mNeonGreen LGG-1(cDNA) let858 3'UTR]; *dyn-1(ky51ts)*	6
RT4575	pwSi161[Pmec-7 mNeonGreen LGG-1(cDNA) let858 3'UTR]; *epg-8(bp251)*	6
RT4486	pwSi161[Pmec-7 mNeonGreen LGG-1(cDNA) let858 3'UTR]; *epg-6(bp242); him-5 (e1490)*	6
RT4487	pwSi161[Pmec-7 mNeonGreen LGG-1(cDNA) let858 3'UTR]; *epg-1(bp414)*	6
RT4427	pwSi222 [Pmec-7 LMP-1 mScarleti let858 3'UTR]; pwSi345[Pmec-7 clic-1 mNeonGreen let858 3'UTR]	8
RT4428	pwSi222 [Pmec-7 LMP-1 mScarleti let858 3'UTR]; pwSi345[Pmec-7 clic-1 mNeonGreen let858 3'UTR]; *rme-8(b1023ts)*	8
RT4604	pwSi222 [Pmec-7 LMP-1 mScarleti let858 3'UTR]; pwSi345[Pmec-7 clic-1 mNeonGreen let858 3'UTR]; *rme-8(pw22[N861S])*	8
RT4593	pwSi222 [Pmec-7 LMP-1 mScarleti let858 3'UTR]; pwSi345[Pmec-7 clic-1 mNeonGreen let858 3'UTR]; *snx-1(tm847)*	8
RT4594	pwSi222 [Pmec-7 LMP-1 mScarleti let858 3'UTR]; pwSi345[Pmec-7 clic-1 mNeonGreen let858 3'UTR]; *bec-1(ok691) IV/nT1 [qIs51]*	8
RT4595	pwSi222 [Pmec-7 LMP-1 mScarleti let858 3'UTR]; pwSi345[Pmec-7 clic-1 mNeonGreen let858 3'UTR]; *vps-15(or1235ts)*	8
RT4636	pwSi495[Pmec-7 RME-8oxGFP let858]; pwSi499 [Pmec-7 clic-1 mScarleti let858]	8
